# Making Sense of Information Overload: Consumer Ranking of Nutritional Claims in Cereal Based Products

**DOI:** 10.3390/nu11122858

**Published:** 2019-11-21

**Authors:** Azucena Gracia, Jesús Barreiro-Hurlé

**Affiliations:** 1 Unidad de Economía Agroalimentaria y de los Recursos Naturales, Centro de Investigación y Tecnología Agroalimentaria de Aragón (CITA), 50059 Zaragoza, Spain; 2 Instituto Agroalimentario de Aragón—IA2, CITA-Universidad de Zaragoza, 50059 Zaragoza, Spain; 3 European Commission, Joint Research Centre (JRC), 41092 Seville, Spain; jesus.barreirohurle@gmail.com

**Keywords:** consumer preferences, nutritional labels, biscuits, pastries, Aragón

## Abstract

As a result of increased consumer awareness, demand for healthier food products is increasing day by day. Consumers seek healthier versions of food products which they relate to reduced presence of unhealthy components or increased presence of healthy ones. As a result, the food industry has not only increased the variety of products available but also uses nutritional claims to signal the presence of more substances. As an average consumer at the supermarket devotes just a few seconds to selecting each product, they are only able or willing to process that information that immediately attracts their attention or that is felt to be more important to them. This paper analyses how consumers rank different nutritional claims for two processed cereal products. Five claims were chosen to reflect the current market landscape of availability, and that relates to both “healthy” (i.e., fiber) and “unhealthy” (i.e., fat) substances. We use a direct ranking preference method with data from a survey conducted with consumers in a Spanish region in 2017. Results show that the ranking of claims differs between the two products (biscuits and pastries) and across consumers. However, consumers prefer those that show reduced presence of unhealthy substances above those that highlight the presence of healthy ones. Therefore, policy to maximize the impact of nutritional labelling should be product-specific.

## 1. Introduction

Consumers’ awareness of the healthiness of foods is increasing and as a result they are starting to demand healthier food alternatives. Industry responds to this demand by offering variants that reduce or eliminate unhealthy components (such as fat) and/or add beneficial ingredients (such as fibre) thereby increasing the presence of food products that carry one or more nutritional claims on supermarket shelves. Results from a survey of the presence of claims on food products stated that food products normally provide more than one claim, either by repeating the claim several times on the package or including claims on two or more substances [1]. The latter happens because food products have been produced with added or removed substances (e.g., ‘high in fibre’, ‘low fat’). In the review reported it was also noted that 21% of food products in the market carried at least one nutritional claim and they often carried multiple claims, in particular, the average number of nutritional claims carried by the product was two in the European countries and 2.1 in Spain. This increased diversity in supply contrasts with the limited time consumers spend in the store to select a food product from the aisle, on average, between 23 and 29 s [2,3]. The presence of multiple claims can lead to a situation of information overload which complicates the selection of healthy food in a supermarket and, as a response, the average consumer generally reverts to a heuristic which involves routinely picking out the same product in few seconds [3]. This routine selection of products implies that consumers are only able or willing to process the information that attracts their attention or that is perceived to be more important for them [4]. Against this landscape of multiple claims per product, it is clear that both from a food industry and a public health perspective it is necessary to understand which nutritional claims drive consumer choices. This is the main objective of this paper.

In particular, the importance attached by consumers to several nutritional claims on processed cereal products was assessed. The selection of the processed cereal products to be analysed and the nutrients to be claimed is based on the results of existing literature reviews on the prevalence of nutritional claims in the market and previous empirical papers on consumers and nutritional claims. Most of these reviews study only a single country being respectively the UK, Ireland, Slovenia, and Spain [5,6,7,8]. The only multi-country review [1] covers Germany, the Netherlands, Slovenia, Spain, and the United Kingdom. This review concludes that for the five countries surveyed the food category with a higher prevalence of nutritional claims is that of cereals and cereal products (31%) closely followed by dairy products (28%). Our application focuses on two products from the cereal category. This review also stated that apart from minerals and vitamins, the most frequent nutritional claims were related to fat content (24%), sugar (12%), and fiber (9%) [1]. In addition, previous research on consumers identify that the most important nutritional claims for them are those related to the content of fat, fiber, and sugar while the level of interest is lower for salt in Italy [9]. However, in Spain, consumers attach the highest importance to the fat-free and source of calcium claims and the least to the low sugar in the case of yogurts [10]. Similarly, a more recent research stated that apart from the health claims, consumers gain the highest utility from the high in fiber and fat-free nutritional claims [11]. Considering the high availability of nutritional claims, we selected several types of claims related to fat, fiber, sugar, and salt.

The importance given by consumers to the selected claims when shopping two based cereal products was assessed using a direct ranking preference method with primary data from a survey administrated to consumers in a region of Spain in 2017. As prior research has identified that preferences for nutritional claims are heterogeneous across consumers [12,13,14,15,16] and products [17], the specification of the model accommodates differences in preferences across both domains.

## 2. Materials and Methods

### 2.1. Product Selection

A field survey of cereal products (biscuits, pastries, breakfast cereals) available in different supermarkets in the region was undertaken to compile the available nutritional claims at the time this study was carried out. The information on cereal products was gathered by direct observation of the aisles of main supermarkets and hypermarkets in the region. Based on the results of this survey, two specific cereal products were chosen. First, biscuits were selected as the product category with the highest prevalence of nutritional claims. A second product category, pastries, was chosen as it is a close substitute of the biscuit category but with a lower perceived level of healthiness by consumers. Last, we needed to select the type of claims related to the substances previously specified (fat, fiber, sugar, and salt). We selected three prevalent nutritional claims for the cereal products (source of fiber, reduced fat, and with no added sugar), and one less prevalent (reduced saturated fat). In addition, although salt content was of less value by consumers, the “low salt” claim was also included based on the recommendation for low salt intake in vulnerable consumers groups (children and elderly).

### 2.2. Data Collection and Survey

Information on consumers was collected using an online survey administrated in the Spanish region of Aragon (a NUTS2 region in North-eastern Spain) in 2017. The target population was established as people living in the region older than 18 years mainly responsible for food purchase. This region was selected as its socio-demographics are similar to those of the Spanish Census of Population (Table A1 in the Appendix A), then, considered to be representative of the Spanish population. Consumers were stratified by gender, age, and province of residence (Aragon is a NUTS2 region which is composed of 3 NUTS3 provinces (Huesca, Teruel and Zaragoza).). Sample size for an error of ± 5%, (95.5% (*k* = 2), and *p* = *q* = 0.5 was established at 400. The questionnaire design builds on the authors’ prior experience with nutrition and food consumption questionnaires and was structured in four parts. First, consumers where asked about their food consumption and purchase habits (i.e., How often do you eat each of the following food product? How many meals per day do you eat?). Second, direct rank questions were asked. In the third part, questions on nutritional information behavior and knowledge, and on food diet and health were included (i.e., Do you pay attention to the nutritional information on food products when shopping? Do you read nutritional information on food products? How strong do you consider your nutritional knowledge to be? What do you believe your health status to be? How much impact do you believe your food intake has on your health?). Finally, a set of questions on socio-demographic characteristics were asked (relating to sex, age, education level, income level, province of residence). Before the final questionnaire was distributed additional validity and reliability tests were undertaken. First, the questionnaire was discussed in a focus group of five consumers and, second, a pilot survey was conducted with a sample of respondents (*n* = 15) to test for understanding and response time. The feedback gathered in the focus group and the analysis of the pilot survey concluded that consumers had a clear understanding of what was being asked of them and that they faced no major difficulties when answering. The final survey was conducted by a market research company hired to provide a representative sample of food consumers stratified by gender, age, and province of residence from their on-line panel. The company sought informed consent from participants before they responded to the questionnaire.

### 2.3. Utility Theory Framework

A direct ranking approach was used where respondents have to rank the analyzed nutritional claims from most to least preferred. In order to do so, they were presented a list of nutritional claims and give the preference ranking (from 1; most preferred; to 5; least preferred) for both product categories.

With the ranking information, two aggregate indicators of importance can be calculated, probability of ith rank (for i ranging from 1 to 5), and ranking means. These indicators provide the ordering of preferences, but they are not able to deal with heterogeneity across respondents because of their aggregate nature. To overcome this, rank ordering can be broken into several choice sets taking some ad hoc assumptions. The new data obtained is transformed into a sequence of choice behavior that can be used to estimate any choice model that incorporates the possibility of addressing heterogeneity in preferences (mixed logit). Therefore, consumer preference heterogeneity can be investigated.

As one of our objectives was to understand individual heterogeneity in claim ranking, besides estimating probabilities and rank means which would reflect average consumer preferences, ranking data were re-coded by considering each rank as a sequential process where respondents take a discrete choice between options to estimate individual preferences. Consumers’ ranking were broken down into sequences of choices and a rank-ordered mixed logit was estimated [18]. The estimated parameters of the importance of the analyzed nutritional claims for each of the respondents were utilized to segment consumers into homogenous groups. These consumer segments were characterized using consumption habits, nutrition information behavior and knowledge, food diet and health and personal consumer characteristics.

When faced with a direct ranking question, respondents rank several alternatives from the most to the least preferred. This ranking is assumed to be based on the utility that respondents get from each option. This gathered information can be used within the utility theory framework defined by the random utility model (RUM) assuming that each individual n faces a choice among J options, and he/she gains utility (Unj) from choosing option j over other options. To transform the direct ranking into specific choices the original ranking information of the different options must be transformed into “pseudochoices” or “pseudo-observations”. Thus, for the first pseudo-observation, the choice set includes J options (in our case 5, one for each of the claims), and the dependent variable corresponds with the option ranked as the most important; for the second pseudo-observation, the option ranked first is discarded, leading to a choice set composed of *J*−1 options, and the alternative ranked second becomes the chosen alternative. The process continues until the choice set consisted of only two options. Then, the ranking of J options can be represented as *J*−1 independent choices, and the new dataset includes *J*−1 choices for each respondent [18].

Utility (*U_nj_*) under the random utility model (RUM) has two parts: One, observed by the researcher (*V_nj_*) and the other unobserved and random (*ε_nj_*) distributed i.i.d. extreme value (as for a logit model). According to Lancaster’s model, consumer utility from a product can be split into attribute specific partial utilities, in our case the characteristics from which consumer derives utility are the nutritional claims included in the direct ranking question [19]. To take this into account the utility function is represented as:

(1) $$U_{nj}=\;\beta {{}^{\prime }}_{n}X_{nj}+\varepsilon_{nj},$$

where $\beta {{}^{\prime }}_{n}$ is a vector of parameters of the exogenous variables *X_nj_*, and *ε_nj_* is an independent identically distributed (i.i.d.) error term over individual and options. The $\beta {{}^{\prime }}_{n}$ coefficients for each individual take into account the heterogeneity in preferences. In other words, the vector of parameters $\beta {{}^{\prime }}_{n}$ is random, with a density *g*(*β*/*θ*) where *θ* represents the parameters of the distribution (i.e., mean and standard deviation).

### 2.4. Rank-Ordered Mixed Logit

Within this framework, a mixed rank-ordered logit (MRL) is estimated with the new data from the ranking observations taking into account heterogeneous preferences.

Under the assumptions of a standard logit, the probability of individual n ranking *J* options from most to least important as *j*_1_;…; *j_m_*;…; *j_J_*, where jm represents the option chosen at the ranking order *m*, can be expressed as the product of logit choice probabilities:

(2) $$\mathrm{Pr}ob(ranking\;j_{1},\ldots ,j_{m},\ldots ,j_{J})={\mathrm{Pr}ob(U}_{j_{1}}>\ldots >U_{j_{m}}>\ldots >U_{j_{J}})=\overset{J-1}{\underset{m=1}{\Pi }}\frac{e_{}^{V_{njm}}}{\sum_{k=m}^{J}e_{}^{V_{njm}}}.$$

This expression represents the probability for an individual n of choosing a specific ranking conditional on *β*. The unconditional probability is the integral of that product of probabilities over the density of *β*:

(3) $$\mathrm{Pr}ob(ranking\;j_{1},\ldots ,j_{m},\ldots ,j_{J})=\int \overset{J-1}{\underset{m=1}{\Pi }}\frac{e_{{}_{}}^{V_{nj_{m}}}}{\sum_{k=m}^{J}e_{}^{V_{njk}}}\times g(\beta \left|\theta )d\theta \right..$$

Equation (3) is estimated using the new transformed data described above where the *J*−1 pseudo-observations for each ranking are considered as *J*−1 choices in a panel. The mixed logit takes into account that each respondent has his own coefficients that affect his ranking in the way that the pseudo-observations are correlated [18]. To estimate the rank-ordered mixed logit the Nlogit software was used.

### 2.5. Preference Heterogeneity

Estimated parameters for the rank-ordered mixed logit for each of the respondents ($\beta {{}^{\prime }}_{n}$) are utilized to segment them using a cluster k-means approach. These segments are characterized by consumption habits, nutritional information behavior and knowledge, food diet and health and consumer socio-demographics characteristics defined in Table 1 and Table 2. This characterization was done using chi-square or Bonferroni test [20], for discrete and continuous variables respectively, to test whether statistically significant differences across segments exist for the different characterization variables. Descriptive and bivariate statistics were calculated using STATA software.

## 3. Results

The final sample consisted of 400 consumers and the summary statistics for its characteristics together with some of the general population’s characteristics are shown for comparison in Table 1.

The sample is representative of the population in terms of age, sex, and province of residence. Our sample’s average age is 48 years and half of respondents were female (50%). Table 2 shows that 15% of respondents consumed pastries, cookies, and cakes either daily or never, respectively. In addition, half of the respondents consume those products less than once a week (31.0%) or once week (22.2%). Only a minority of respondents admitted to snacking often (9%) (This figure has to be taken with caution as snacking is considered a non-desirable behaviour and when asked directly for these under-reporting is common [24]) and 17% declared that they never snack. Half of the respondents stated that they pay attention to nutritional information on food packages but only 20% of them always read this information.

Respondents’ self-reported nutritional knowledge is not very high (2.8 in a scale of 5) and they reported a high awareness of the impact their diet had on their health (4.3 in a scale of 5). Around half of the respondents declared that they follow a healthy or very healthy diet (48.8% and 3.5%, respectively). Finally, most of respondents believe that they are healthy or very healthy with only 2% indicating that their health is bad or very bad.

### 3.1. Attribute Importance Ranking

Table 3 and Table 4 show the percentage of respondents that ranked the different nutritional claims in the different levels, together with their mean of the ranks for biscuits and pastries, respectively. It is worth recalling that due to the scale used (order from most important to least important) a lower value of the mean indicates the highest importance for the claim.

Looking at the probability of ranks it can be concluded that the most and least important claim is the same for both product categories “reduced saturated fat” and “low salt”, respectively. Heterogeneity is found for the claim ranked second as for biscuits, that is “source of fiber” while for pastries it is “with no added sugar”. It seems that for the less healthy product (pastries) consumers place more importance on avoiding unhealthy ingredients while for the reference product (biscuits) the importance is placed on healthy components. However, if we focus on the rank means ordering homogeneity is found for the two most important claims (“reduced saturated fat” and “with no added sugar”) which cannot be considered different based on a paired sample *t*-test.

To identify heterogeneity across products, we test whether the mean importance for the different claims differ between the two tested products and we found that only for the claim “with no added sugar” the rank means were statistically the same between biscuits and pastries (last column in Table 5). For the remaining claims, the rank means were statistically different. In particular, the rank means for “source of fiber” and “low salt” were higher for pastries, indicating that the preference for these claims was lower for pastries compared to the biscuits. On the other hand, the rank means for “reduced saturated fat” and “reduced fat” were higher for breakfast biscuits than those for pastries, therefore, these claims are preferred in the case of pastries. This pattern shows that for unhealthy products claims signaling a lower presence of unhealthy components play a greater role in the purchase decision, while for the reference product the presence of healthy substances are more valued.

### 3.2. Consumers Heterogeneity

As mentioned above, we expected that heterogeneity in the ranking of importance of different claims would exist not only between products but also between individuals. Looking at the results reported in Table 3 and Table 4 this heterogeneity becomes evident as the standard deviations of the means rankings are quite high. To gain additional insights on the drivers of this heterogeneity, a ranked-order mixed logit was estimated using NLOGIT 5.0 (Econometric Software INC, Plainview, USA) assuming random parameters following a normal distribution. To avoid the issue of multicollinearity the least preferred nutritional claim (“low salt”) was considered the reference level and excluded in the final specification.

Table 6 shows the mean and the standard deviation of the estimated parameters for the ranked-order mixed logit. The standard deviations of the estimated coefficients were statistically significant at the 1% significance level, meaning that the consumer importance attached to the different claims were heterogeneous. The mean of the estimated parameters were positive and statistically significant indicating that the importance attached to the included nutritional claims were statistically different from the importance given to the reference claim (“low salt”). These estimations corroborate findings based on aggregate rankings reported in Table 3 and Table 4 as the most preferred nutritional claims remained “reduced saturated fat” and “with no added sugar” for both products. The least preferred claims were “source of fiber” and “reduced fat” which occupies the third and fourth position, respectively in the case of biscuits and the fourth and third in the case of pastries. However, the ranked-order mixed logit model provides statistically significant differences across participants’ estimated coefficients.

The estimated parameters for each of the participants ($\beta {{}^{\prime }}_{n}$) were used to segment them into homogeneous consumers groups using a k-means cluster analysis [20]. From the cluster analysis, we obtained four segments for both products (Table 7). The size of the clusters for the two products can be seen in the first two rows in Table 7 and the mean values of the estimated parameters for each cluster in the subsequent rows.

The latter mean values were used to name the different clusters using the profile of the claim importance. For biscuits, we observed that consumers in the first two clusters attached the least importance to the “low salt” claim while the other two clusters have different least important claims: Reduction of fat (“reduced saturated fat” and “reduced fat”) for cluster 3 and “source of fiber” for cluster 4. Based on the least preferred claim we named clusters 3 and 4 “fat careless” and “fiber careless”. Then, because cluster 1 showed the highest preference for the “source of fiber” claim it was named “fiber lovers”, despite the lower preference for “low salt”. Similarly, as cluster 2 showed the highest preference for the “reduced saturated fat” closely followed by the “reduced fat” claim this cluster was named “fat avoiders”. For pastries, we also observed that two clusters (cluster 2 and cluster 3) attached the least importance to the “low salt” claim while the other two clusters attached less importance to reduction in fat content (cluster 4 “reduced saturated fat” and “reduced fat”) and “source of fiber” (cluster 1). These last two clusters were named “fat careless” and “fiber careless”. On the other hand, as cluster 2 attached the highest preference for the “reduced saturated fat” followed by the “reduced fat” claim this cluster was named “fat avoiders”. For cluster 3, the only distinguishing preference pattern is showing the lowest preference for the “low salt” claim while the estimated means for the rest of the claims were not much different, therefore we named “salt careless”.

### 3.3. Segments Profiling

Table 8 and Table 9 shows the chi-square and ANOVA (Bonferroni) test results among the four segments and the consumers’ characteristics that will allow profiling of the clusters. In particular, the consumers’ characteristics displayed in Table 1 and Table 2 that were found statistically different across clusters at least at a 10% significance level are included in the tables.

For breakfast cereals, Table 8 shows that the “fiber lovers” and the “fat avoiders” include a higher proportion of young and female individuals than the other two segments. In addition, a larger proportion of consumers in these segments declared following a healthy or very healthy diet (63.9% for “fiber lovers” and 56.4% for “fat avoiders”) in comparison to the other two segments (48.6 and 42.2, respectively). Thus, it seems that there is some correlation between the importance given to claims signaling healthier product versions and healthy dietary habits.

The main difference between these clusters is that a higher proportion of consumers in the “fat avoiders” segment claimed eating pastries, cookies, and cakes on a daily basis. In contrast, consumers in the segments “fat careless” and “fiber careless” were characterized by being older males with an average perception of following a healthy diet. The main difference between these two clusters was that a higher proportion of consumers in the “fiber careless” cluster stated that they never eat pastries, cookies, and cakes (22.4%).

For pastries, Table 9 shows that “fat careless” consisted of a lower proportion of older females. In addition, a higher proportion of consumers in this segment state that they exercise (75%) and never eat pastries cookies and cakes (20%) in comparison with the other three clusters. We observed similar personal characteristics of consumers in this cluster and the segment “fiber carless” namely that of the proportion of females, the frequency of consumption of pastries, cookies and cakes, and the perceived impact of the food diet on health. On contrary, the “fat avoiders” segment consisted of a higher proportion of females, the youngest among clusters, and living in bigger households. Consumers in this cluster also presented with the highest awareness that food diet has an impact on health. In addition, the “fat avoiders” segment consisted of the lowest proportion of people that exercise and the lowest proportion of consumers that never eat pastries, cookies, and cakes. Finally, the “salt careless” segment consisted of a higher proportion of females living in households of the smallest size. Consumers in this cluster also presented with a high awareness that food diet has an impact on health and consisted of a high proportion of people that exercise.

## 4. Discussion

This study aimed to assess the importance consumers attached to several nutritional claims related to the most prevalent claimed nutrients, some of them beneficial (fiber) and others harmful to health (saturated fat, sugar, fat, and salt). The selected food carriers for the claims were biscuits and pastries because of their different perceived healthiness. Finally, a highly prevalent claim for a beneficial nutrient (“source of fiber”), highly prevalent claims for harmful nutrients (“reduced fat”, “with no added sugar”), and less prevalent ones for harmful nutrients (“reduced saturated fat” and “low salt”) were selected.

The results indicated that for the average consumer the most important nutritional claims for the two cereal products were “reduced saturated fat” and “with no added sugar”. On the other hand, the least important claim was “low salt”. The importance given to the other two claims “source of fiber” and “reduced fat” differs between cereals products. While “source of fiber” is more important than “reduced fat” for biscuits, the opposite was found to be true for pastries. This finding is consistent with previous results for cheese where the “reduced fat” claim is positively valued while the “low salt” negatively [14]. In addition, on breakfast biscuits consumers positively value the claims “high in fiber” and “reduced saturated fat” [15], likewise on cheese the valuation for the low saturated fat claim was higher than the valuation for the low fat claim [25]. On the contrary, a study using a hedonic prices approach found no market valuation for the nutritional claims related to fat, sugar and fiber in the case of yogurts [26]. In particular, they stated that market prices for food products with the nutritional claims “fat free”, “no sugar”, and “source of fiber” are not statistically different from food products without these claims. In addition, we find that preferences differ across consumers, and four segments of consumers were identified based on the estimated importance for the different nutritional claims. Except for one, segments did not differ between biscuits and pastries. These segments were named “fat careless”, “fiber careless”, and “fat avoiders”. In the case of biscuits, a further segment was identified as “fiber lovers” and in the case of pastries, “salt careless”. Heterogeneity and different consumers’ segments according to the consumers’ valuation for nutritional claims were also found for breakfast biscuits and yogurt, respectively [13,15]. In particular, for biscuits, two segments of consumers named “reduced saturated fat lovers” and “fiber lovers” were found because consumers’ valuation for the reduced saturated fat claim was higher than that of the valuation of high in fiber in the “reduced saturated fat lovers” segment and the contrary for the “fiber lovers” [15]. In the case of yogurt also two clusters were detected where consumers preferred yogurt with low or medium sugar content but they differed in the preference for fat content [13]. While one preferred yoghurt with the lowest fat content, the other preferred yoghurt with high fat content. The third segment also preferred yoghurt with the lowest fat content but high in sugar. Our segments also follow this pattern, with some consumers preferring claims that signal negative substances and other that prefer the signal of positive substances. In addition, we find some groups of consumers do not care about the different substances evaluated (fiber careless, salt careless).

As for the characterization of the consumers in the different clusters, the “fat avoiders” segment includes younger females and a more intense consumption of pastries, cookies, and cakes. Whereas, the “fiber careless” segment is characterized by being older males with a higher proportion of households that never consume pastries, cookies, and cakes. In addition, the “fat careless” segment is also characterized by younger females but with a higher proportion of households that never consume pastries, cookies, and cakes.

An interesting result is there are no differences between clusters for education, weight, and health status of individuals, eating habits (snacking), or use of nutritional information. Similar results were also found for Spain where no differences between obese, overweight, and normal weight consumers were detected in the valuation of “high in fiber” and “reduced saturated fat” claims for breakfast biscuits [15]. This result indicates that the weight status itself did not affect the preferences for nutritional claims in the case of cereal products. However, for hard cheese, the valuation of low salt and reduced fat nutritional claims was different for differences in education, income, and body mass index levels [14]. This corroborates that the importance of different claims are product-specific.

Our findings are relevant for food producers and from a public health perspective. Food producers that want to increase the attractiveness of their products for consumers should focus on highlighting the absence, or low levels, of unhealthy substances (fats) for cereal products. This is particularly relevant as consumers with a high consumption of these products attach more importance to these claims. However, this will not stimulate consumption in non-consuming segments, as they tend to place little importance on these claims. From a public health perspective, these findings raise some concerns with regards to moral licensing and the consumption of the less healthy cereal product (pastries) as those consumers that attach more importance to avoidance claims (low fat) tend to consume more products whilst also following a less healthy lifestyle with less exercise. If public authorities and consumer organizations want to avoid that claims are used as an excuse to follow less healthy diets and lifestyles, promotion activities emphasizing the importance of limiting the consumption of these products, even in their healthier versions, are needed. This seems to be a general trend for less healthy products (sausages) [14]. Based on the profile of the “fat avoider” cluster, these promotional initiatives should target only male and younger consumers, communicating them that, although they do not have yet health problems related to their diet, following a healthy diet is the best way of preventing.

Finally, we would like to highlight the strengths and limitations of our study. Regarding the former, our sample is an exact representation of the target population for all age ranges, however it underrepresents respondents with secondary education with a bias towards the more educated strata. This bias is common in the majority of studies using primary data as this strata is more disposed to respond to questionnaires [27]. Moreover, the sample is also representative of the population in terms of the Body Mass Index (BMI) as the percentage of our sample of consumers belonging to the three BMI groups was very similar to the percentage in the general population. In addition, we have shown how claim valuation changes within a specific product category between healthy and unhealthy products. As for limitations, while our results hold for the specific population and products selected, the generalization of the results are not straightforward. While some of the socio-economic and behavioral drivers seem to be constant with regards to the valuation of nutritional claims, the implications regarding consumption can be population specific. More studies are needed to confirm that nutritional claims in less healthy products can be driving moral licensing for their consumption. Moreover, the study of consumers’ importance for the nutritional claims was applied only to two food products within a specific food product category (cereals). As preferences for nutritional claims were found to be product-specific, conclusions and recommendations might vary when applied to other food categories.

## 5. Conclusions

The ranking of nutritional claims varies across consumers and differs between the two products (biscuits and pastries). Consumers prefer the nutritional claims that show reduced presence of unhealthy substances above those that highlight the presence of healthy ones. For the average consumer, the most important nutritional claims for the two cereal products were “reduced saturated fat” and “with no added sugar” while the least important claim was “low salt”. The importance given to the other two claims “source of fiber” and “reduced fat” differs between cereals products. While “source of fiber” is more important than “reduced fat” for biscuits, the opposite was found to be true for pastries. Four segments of consumers were identified based on the estimated importance for the different nutritional claims. Except for one, segments did not differ between biscuits and pastries. These segments were named “fat careless”, “fiber careless”, and “fat avoiders”. In the case of biscuits, a further segment was identified as “fiber lovers” and in the case of pastries, “salt careless”. The characterization of the segments indicate that the “fat avoiders” includes younger females and a more intense consumption of pastries, cookies, and cakes. Whereas, the “fiber careless” segment is characterized by being older males with a higher proportion of households that never consume pastries, cookies, and cakes. In addition, the “fat careless” segment is also characterized by younger females but with a higher proportion of households that never consume pastries, cookies, and cakes.

## Figures and Tables

**Table 1:** Sample demographic characteristics (%, unless stated).

Characteristics	Sample (*n* = 400)	Population
Gender ^a^		
Male	50.0	49.4
Female	50.0	50.6
Age ^a^ (average, standard deviation)	48.0 (14.0)	N/A
18–34	21.0	21.1
35–44	21.0	19.1
45–54	19.9	18.6
≥ 55	38.5	41.3
Studies level b		
Primary	27.5	24.6
Secondary	32.5	50.0
Higher	40.0	25.4
Income range		
≤ 1000 €/month	10.0	N/A
1001–2500 €/month	42.2	N/A
2501–4500 €/month	17.5	N/A
> 4500 €/month	3.3	N/A
Do not know/refuse to answer	27.0	N/A
Household size (average, standard deviation)	2.7 (1.1)	N/A
Province of residence ^a^		
Huesca	14.2	17.0
Teruel	6.8	11.0
Zaragoza	79.0	72.0
Body Mass Index (BMI) ^c^	25.8 (4.6)	N/A
Less than 25	48.2	47.5
25–30	35.7	38.8
More than 30	16.1	15.7
Practice exercise or walk more than 30 min at least five times a week	66.5	N/A

^a^ Instituto Nacional de Estadística, INE (2017a) [21]; ^b^ Instituto Aragonés de Estadística, IAEST (2018) [22]; ^c^ INE (2017b) [23]. N/A: not available.

**Table 2:** Food consumption and health habits (%, unless stated).

Consumption Habits	
Number of meals (average)	3.7 ± 0.94
Frequency of consumption pastries, cookies, and cakes	
Never	14.8
Less than one a week	31.0
Once a week	22.2
Several times a week	16.2
Daily	15.8
Snacking	
Never	17.2
Sometimes	73.8
Often	9.0
Nutritional information	
Pay attention (yes)	49.0
Read always (% of those paying attention)	20.4
Nutritional knowledge (average)^a^	2.8 ± 0.93
Diet and health	
Perceived impact of food diet on health (average) ^a^	4.3 ± 0.74
Follow a healthy diet	
Very unhealthy diet	0.5
Unhealthy diet	6.2
Neutral	41.0
Healthy diet	48.8
Very healthy diet	3.5
Self-reported health status	
Very unhealthy	0.5
Unhealthy	1.5
Neutral	21.3
Healthy	66.7
Very healthy	10.0

^a^ In a 5 point increasing scale where 1 indicates the lowest level and 5 the highest.

**Table 3:** Probability of ranks and ranking means: Biscuits.

	Rank #1 (%)	Rank #2 (%)	Rank #3 (%)	Rank #4 (%)	Rank #5 (%)	Mean ± SD
Source of fibre ^a^	30.3	15.5	16.5	16.2	21.5	2.83 ± 1.54
Reduced saturated fat ^b^	30.8	21.0	21.2	14.2	12.8	2.57 ± 1.38
With no added sugar ^b^	27.2	23.8	21.5	19.2	8.3	2.57 ± 1.29
Reduced fat ^c^	6.5	26.8	24.5	23.2	19.0	3.21± 1.21
Low salt ^d^	5.2	13.0	16.2	27.0	38.5	3.80 ± 1.23

^a,b,c,d^ Superscript letters mean that importance means are statistically different among nutritional claims using the *t*-test. Note: Rank #1 indicates ranked as the most important (in the first position).SD, Standard Deviation.

**Table 4:** Probability of ranks and ranking means: Pastries.

	Rank #1 (%)	Rank #2 (%)	Rank #3 (%)	Rank #4 (%)	Rank #5 (%)	Mean ± SD
Source of fibre ^a^	21.2	11.8	19.2	23.5	24.3	3.17 ± 1.46
Reduced saturated fat ^b^	41.0	24.2	12.3	13.2	9.3	2.25 ± 1.35
With no added sugar ^b^	25.2	24.3	25.2	16.5	8.8	2.59 ± 1.27
Reduced fat ^c^	8.0	31.0	26.2	21.5	13.3	3.01 ± 1.17
Low salt ^d^	4.5	8.8	17.0	25.2	44.5	3.96 ± 1.17

^a,b,c,d^ Superscript letters mean that importance means are statistically different among nutritional claims using the *t*-test. Note: Rank #1 indicates ranked as the most important (in the first position).

**Table 5:** Test of differences for the nutritional claims between biscuits and pastries.

	Breakfast Biscuit	Pastries	Paired *t*-Test (*p*-Value) for Differences between Columns
Source of fibre	2.83 ***	3.17 ***	−5.56 (0.00)
Reduced saturated fat	2.57 ***	2.25 ***	5.23 (0.00)
With no added sugar	2.57 ***	2.59 ***	−0.26 (0.39)
Reduced fat	3.21 ***	3.01 ***	3.31 (0.00)
Low salt	3.80 ***	3.96 ***	−2.75 (0.00)

Note: *** denotes statistical significance at 1% significance level within-columns.

**Table 6:** Estimation results of the rank-ordered mixed logit.

	Biscuits		Pastries	
Parameters Estimates	Coefficient	Z-Ratio	Coefficient	Z-Ratio
Source of fibre	1.7542	4.07 ***	1.1954	3.46 ***
Reduced saturated fat	2.2311	4.31 ***	2.8022	4.11 ***
With no added sugar	2.1990	4.31 ***	2.1017	4.06 ***
Reduced fat	1.1985	3.90 ***	1.6510	4.04 ***
	Standard deviation of parameters			
Source of fibre	3.4485	3.40 ***	2.3215	2.59 **
Reduced saturated fat	3.6425	4.26 ***	3.1126	3.10 **
With no added sugar	2.3897	3.58 ***	1.8733	2.16 **
Reduced fat	2.8731	7.62 ***	2.3041	2.54 **
Number of observations	1600		1600	
Log likelihood (at convergence)	−1749.8		−1694.5	
McFadden Pseudo R-square	0.32		0.34	

Note: ***, ** denotes statistical significance at 1% and 5%, significance levels respectively.

**Table 7:** Segmentation of consumer according to nutritional claim importance.

	Cluster 1	Cluster 2	Cluster 3	Cluster 4
Cluster size (%)				
Breakfast biscuits	27	27	27	19
Pastries	18	27	30	25
Source of fibre				
Breakfast biscuits	4.65 ^a^	1.43 ^b^	2.11 ^c^	−1.88 ^d^
Pastries	−0.94 ^a^	1.17 ^b^	2.49 ^c^	1.18 ^d^
	Reduced saturated fat			
Breakfast biscuits	2.87 ^a^	5.61 ^b^	−1.38 ^c^	1.60 ^d^
Pastries	3.22 ^a^	5.74 ^b^	2.84 ^c^	−0.56 ^d^
With no added sugar				
Breakfast biscuits	2.62 ^a^	2.92 ^b^	1.41 ^c^	1.70 ^d^
Pastries	1.98 ^a^	2.35 ^b^	2.61 ^c^	1.37 ^d^
Reduced fat				
Breakfast biscuits	1.62 ^a^	3.83 ^b^	−1.51 ^c^	0.72 ^d^
Pastries	1.81 ^a^	3.76 ^b^	1.79 ^c^	−0.77 ^d^

^a,b,c,d^ Superscript letters mean that importance means are statistically different among clusters.

**Table 8:** Biscuits: Profiling consumer segments (%, unless stated).

Characteristics	Fiber Lovers	Fat Avoiders	Fat Careless	Fiber Careless	Total Sample
Gender					
Female **	54.2	57.8	46.8	40.2	50.0
Age (average) ***	47.0	44.6	49.1	51.6	48.0
Frequency of consumption pastries, cookies, and cakes (%)					
Never **	14.0	10.1	11.7	22.4	14.8
Daily ***	14.0	25.7	13.0	9.4	15.8
Nutritional knowledge (average) **	2.7	2.6	3.0	2.7	2.8
Follow a healthy diet (%)					
Very unhealthy and unhealthy diet	7.2	11.2	1.9	6.8	6.7
Neutral	28.9	32.4	49.5	51.0	41.0
Healthy diet	61.5	50.9	43.9	41.2	48.8
Very healthy diet	2.4	5.5	4.7	1.0	3.5

Note: ***, ** means statistical significance at 1% and 5%, significance levels, respectively.

**Table 9:** Pastries: Profiling consumer segments (%, unless stated).

Characteristics	Fiber Careless	Fat Avoiders	Salt Careless	Fat Careless	Total Sample
Gender					
Female ***	37.5	61.3	59.8	35.0	50.0
Age (average) ***	47.8	43.6	47.4	53.5	48.0
Household size (average) *	2.6	2.9	2.5	2.7	2.7
Practice exercise or walk more than 30 min at least five times a week **	62.5	57.5	69.7	75	66.5
Frequency of consumption pastries, cookies, and cakes (%)					
Never ***	19.4	5.6	15.6	20.0	14.8
Perceived impact of food diet on health (average) **	4.2	4.4	4.3	4.2	4.3

Note: ***, **, * means statistical significance at 1%, 5%, 10% significance levels, respectively.

## Appendix A

**Table A1:** Population by sex and age in Spain and in the region (%).

	Total	Sex		Age				
		Female	Male	18–34	35–44	45–54	55–64	More Than 64
Spain	46,572,132	51.0	49.0	22.9	20.2	19.0	15.2	22.9
Region	1,308,750	50.6	49.4	21.1	19.1	18.6	15.5	25.8

Source: Spanish Census of Population, 2017. Instituto Nacional de Estadística (www.ine.es), Spain.
